# Leptin Concentration, Obesity, and Plasma Non-esterified Fatty Acid Levels in Children

**DOI:** 10.3389/fped.2021.812779

**Published:** 2022-01-06

**Authors:** Claudia Vales-Villamarín, Henar Ortega-Senovilla, Olaya de Dios, Iris Pérez-Nadador, Teresa Gavela-Pérez, Leandro Soriano-Guillén, Carmen Garcés

**Affiliations:** ^1^Lipid Research Laboratory, Instituto de Investigación Sanitaria-Fundación Jiménez Díaz, Universidad Autónoma de Madrid, Madrid, Spain; ^2^Faculties of Pharmacy and Medicine, Universidad San Pablo-CEU, Madrid, Spain; ^3^Department of Pediatrics, Instituto de Investigación Sanitaria-Fundación Jiménez Díaz, Universidad Autónoma de Madrid, Madrid, Spain

**Keywords:** non-esterified fatty acids, leptin, obesity, BMI, children

## Abstract

The association between obesity and higher non-esterified fatty acid (NEFA) levels has been established in adults. In contrast, lower NEFA levels have been described in children with obesity although the reason behind this association remains unclear. Leptin, which regulates body weight and plays a role in lipolysis, could be involved in this relationship. We evaluated the influence of leptin in the association between obesity and NEFA concentrations in children, analyzing two cohorts including 684 6- to 8-year-olds and 836 12- to 16-year-old children, respectively. After adjusting by leptin, insulin levels remained significantly higher in adolescents with obesity as compared with levels in those without obesity. However, insulin levels showed no differences between prepubertal children with and without obesity. The significantly lower NEFA concentrations observed in 6- to 8-year-old girls with obesity disappeared when comparing NEFA levels between girls with and without obesity after adjusting by leptin. We report an influence of leptin levels on the association between obesity and insulin and NEFA in young children that is not observed in adolescents. Our findings add information about factors that may contribute to explain the lower NEFA levels described in prepubertal children with obesity.

## Introduction

The significant worldwide increase in the prevalence of childhood obesity has been associated with a rise in metabolic complications and in the prevalence of type II diabetes in children (1). In adults, it is accepted that obesity determines the onset of insulin resistance by impairing insulin signaling, and non-esterified fatty acids (NEFA) have been postulated as the link between obesity and insulin resistance (2). Obesity has been associated with insulin sensitivity also in children (3). However, even though obesity in children has been associated to increased NEFA levels in some studies (4, 5), others have failed to find any differences on NEFA by weight category (6) or have described lower NEFA levels in children with obesity (7, 8). In a previous study of our group in prepubertal children, NEFA levels were significantly lower in girls with obesity than in girls without obesity (9). No differences in NEFA concentrations by weight category were found analyzing adolescents (10). Although it seems that the association may depend on age and sex, the reasons for these different findings remain unknown.

Leptin, which plays an important role in the regulation of energy homeostasis and body weight, exerts direct, and indirect effects on adipocyte metabolism, affecting lipogenesis, triglyceride hydrolysis, and fatty acid oxidation (10) and could be involved in the relationship between obesity and NEFA concentrations. An association of leptin with NEFA metabolism has been described already in young children (11).

The aim of our study was to analyze the role of leptin in the association between obesity and NEFA levels in children.

## Methods

### Subjects

Our population-based samples comprise 684 (338 boys, 346 girls) 6- to 8-year-old children and 836 (396 boys, 440 girls) adolescents, aged 12–16 years. These subjects were participants of two previous studies comparing biochemical variables, including insulin and NEFA levels, between children with and without obesity (9, 10). In these cross-sectional studies children were selected by means of random cluster-sampling in schools and stratified by sex and type of school. Unfortunately, no information on Tanner stage is available in our study, but information on age at menarche was available in order to classify the pubertal status of the girls. Nevertheless, all children reported by parents to be suffering from chronic disease, including precocious and delayed puberty, were excluded from the study.

Parents or legal guardians were required to provide written consent for their children to participate in the study. The study protocol complied with the Helsinki Declaration guidelines and was approved by the Clinical Research Ethics Committee of the IIS-Fundación Jiménez Díaz (PIC016-2019 FJD).

### Anthropometric Data

Measurements were taken with the children lightly dressed and barefoot. Height was measured to the last millimeter using a portable stadiometer, weight was recorded to the nearest 0.1 kg using a standardized electronic digital scale. BMI (weight in kg/height in m squared) was calculated from these measurements. Z-score BMI was also calculated according to Spanish reference data. Children were categorized as having obesity according to the age- and sex-specific BMI cut-off points proposed by Cole et al. (12).

### Biochemical Data

Fasting (12-h) blood samples were obtained early in the morning by venipuncture into Vacutainer tubes. A team consisting of one physician and several nurses was in charge of blood extractions and physical measurements. Samples were centrifuged (1,500 g at 4°C for 25 min), and serum and plasma were separated in aliquots, which were immediately stored at −80°C for further analysis.

Insulin concentrations were measured using a commercial kit (BI-Insulin IRMA, Bio-Rad, Marnes la Coquette, France). Non-esterified fatty acid were measured by using the Wako NEFA-C kit (Wako Industries, Osaka, Japan). Leptin concentrations were determined by ELISA using a commercial kit (Leptin EIA-2395, DRG, Marburg, Germany).

### Statistical Analysis

Statistical analyses were performed using the SPSS software package, version 21.0 and GraphPad Prism statistical software, version 8. The normality of distribution of the variables under study was examined using the Kolmogorov–Smirnov test. Spearman correlation and partial correlations analyses were used to evaluate the correlations between the variables under study. Given their skewed distributions, concentrations were log-transformed before statistical comparison. Student's *t*-test was used to analyze differences in NEFA and insulin between subjects with and without obesity by sex and age group. Non-esterified fatty acid and insulin concentrations by weight category, adjusted by leptin, were compared by analysis of covariance (ANCOVA).

## Results

The characteristics of the study participants by group of age and sex are shown in Table 1. The average ages in our study were 7.2 ± 0.6 years in prepubertal children and 14.4 ± 1.1 years in adolescents, without differences between sexes. No differences in BMI and BMI z-score were observed between males and females of any group of age. The prevalence of obesity was 10% in prepubertal children, similar in both sexes, and 7.5 and 4.5% in 12- to 16-year-old boys and girls, respectively. Leptin concentrations were significantly higher in females than in males in both groups of ages.

**Table 1:** Anthropometric and biochemical characteristics (mean ± SD) of males and females by age.

	**6- to 8-year-olds**		
	**Males (*n* = 338)**	**Females (*n* = 346)**	***P***
Age (years)	7.2 ± 0.6	7.2 ± 0.7	0.867
Weight (kg)	26.9 ± 5.2	26.7 ± 5.5	0.698
BMI (kg/m^2^)	16.8 ± 2.3	17.0 ± 2.6	0.530
Z-Score BMI	−0.01 ± 0.95	0.04 ± 1.02	0.497
Insulin (μU/ml)	3.2 ± 2.1	3.7 ± 2.7	0.006
NEFA (mEq/L)	0.66 ± 0.25	0.70 ± 0.28	0.035
Leptin (ng/ml)	3.9 ± 4.9	6.4 ± 7.2	0.000
	**12- to 16-year-olds**		
	**Males (*****n*** **=** **396)**	**Females (*****n*** **=** **440)**	***P***
Age (years)	14.4 ± 1.2	14.4 ± 1.1	0.818
Weight (kg)	62.3 ± 15.0	56.1 ± 10.2	0.000
BMI (kg/m^2^)	21.9 ± 4.0	21.7 ± 3.4	0.318
Z-Score BMI	0.16 ± 1.08	0.14 ± 0.98	0.811
Insulin (μU/ml)	8.7 ± 6.9	8.5 ± 4.8	0.633
NEFA (mEq/L)	0.43 ± 0.20	0.44 ± 0.20	0.455
Leptin (ng/ml)	6.1 ± 8.1	16.0 ± 10.1	0.000

**Figure 1:**
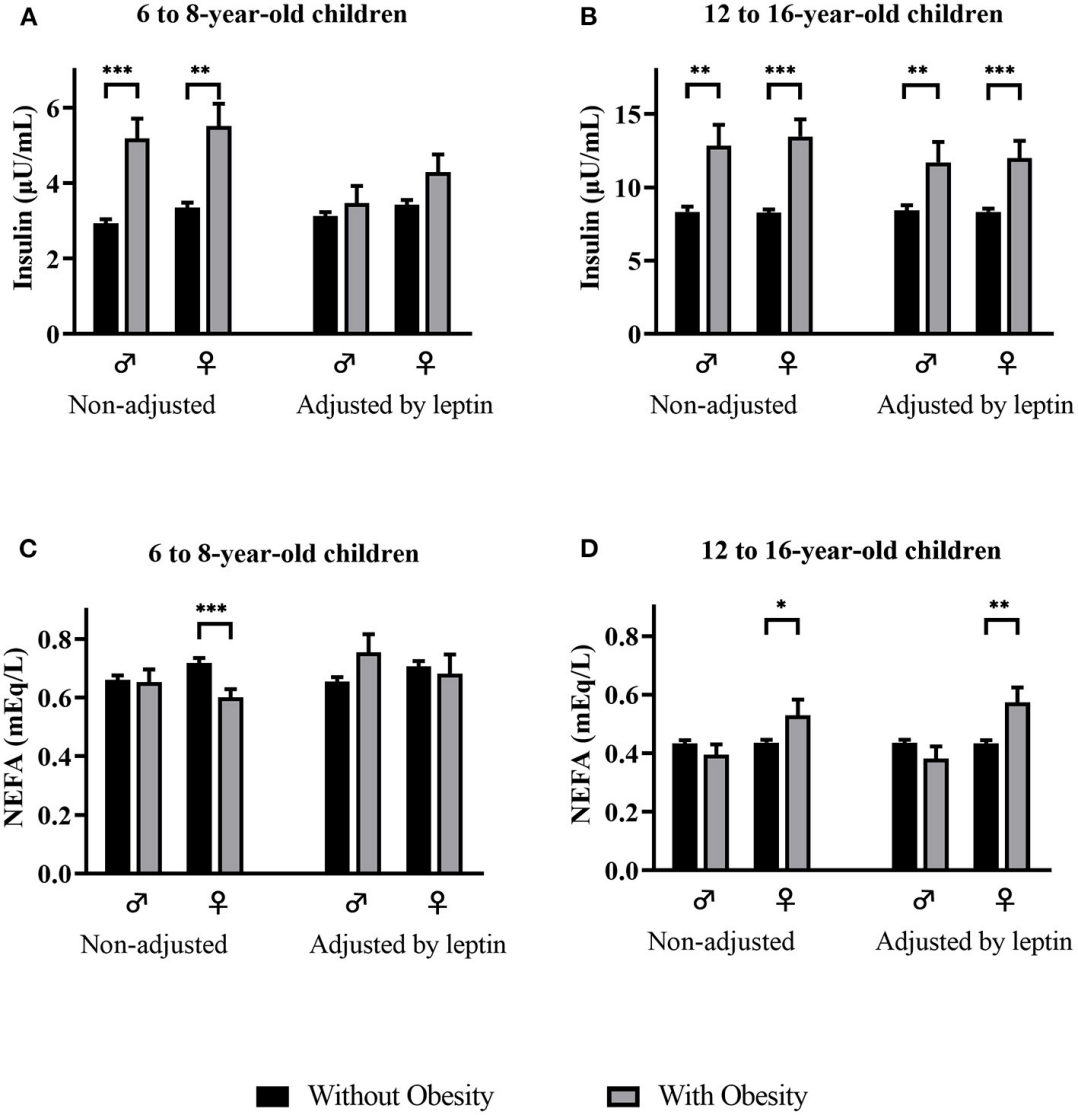
Insulin **(A,B)** and NEFA **(C,D)** levels (mean and std. error) by weight category, non-adjusted and adjusted by leptin levels, in males and females of both age groups. NEFA, non-esterified fatty acids. *p*-value: ANCOVA *p < 0.05; **p < 0.01; ***p < 0.001.

When comparing mean insulin concentrations between children with and without obesity by sex in 6- to 8-year-olds, non-adjusted and adjusted by leptin, (Figure 1A), we observe that the significant differences observed in both sexes disappear after adjusting by leptin levels. On the other hand, in 12- to 16-year-olds, the significantly higher insulin levels observed in males and females with obesity remain significantly higher after adjusting by leptin (Figure 1B). When comparing NEFA by weight category in 6- to 8-year-olds (Figure 1C), we observed significantly lower NEFA concentrations in females with obesity than in females without obesity, but observed no significant differences in NEFA levels by weight category after adjusting by leptin. No differences in NEFA levels by weight category were observed in 6- to 8-year-old males without adjusting or adjusting by leptin levels (Figure 1C). In 12- to 16-year-olds (Figure 1D), higher NEFA levels were observed in females with obesity independently of leptin levels. No significant differences in NEFA concentrations were observed in 12- to 16-year-old males with or without obesity.

Spearman correlation analysis showed a significant positive correlation between leptin and insulin levels in males and females of both age groups (Table 2). On the other hand, leptin concentrations correlated negatively with NEFA in prepubertal children, without showing correlation in adolescents. After adjusting by BMI, leptin levels remain positively correlated with insulin and negatively correlated with NEFA in 6- to 8-year-old children but the correlation disappears in 12- to 16-year-old boys, being only weakly related to insulin in 12- to 16-year-old girls (Table 2).

**Table 2:** Correlation analysis of leptin with insulin and NEFA, non-adjusted and adjusted by BMI in 6- to 8-year-old and 12- to 16-year-old males and females.

	**6- to 8-year-olds**			
	**Leptin (non-adjusted)**		**Leptin (adjusted by BMI)**	
	**Males**	**Females**	**Males**	**Females**
Insulin (μU/ml)	0.384^**^	0.465^**^	0.272^**^	0.293^**^
NEFA (mEq/L)	−0.201^**^	−0.250^**^	−0.125^*^	−0.127^*^
	**12- to 16-year-olds**			
	**Leptin (non-adjusted)**		**Leptin (adjusted by BMI)**	
	**Males**	**Females**	**Males**	**Females**
Insulin (μU/ml)	0.284^**^	0.309^**^	0.046	0.116^*^
NEFA (mEq/L)	0.055	−0.053	0.061	−0.062

* *p < 0.05;*

** *p < 0.001*.

## Discussion

In previous studies, we described that males and females with obesity had higher plasma insulin levels and HOMA values than their non-obese counterparts in prepubertal (9) as well as in 12- to 16-year-old children (10). However, contrary to observations in adults, no significant differences by weight category were found in NEFA levels in 12- to 16-year-old children or in 6- to 8-year-old boys (9, 10) and significantly lower NEFA levels were found in 6- to 8-year-old girls with obesity comparing with girls without obesity (9). In the current study, we describe that leptin is influencing the association of obesity with NEFA and insulin levels in prepubertal children, since we observed that, after adjusting by leptin, the differences in NEFA and insulin concentrations between 6- to 8-year-old children with and without obesity disappear. On the contrary, no influence of leptin levels on the differences in NEFA and insulin concentrations between children with and without obesity was observed in 12- to 16-year-old children. Furthermore, we observed that leptin, highly related to BMI, correlated positively with insulin in both age groups and negatively with NEFA in prepubertal children. However, after adjusting by BMI, the correlations disappeared in adolescents while remained significant in prepubertal children.

It has been suggested that leptin has an important influence on NEFA metabolism (11, 13) and that changes in adipokines secretion may precede changes in plasma NEFA concentrations (8).

Leptin concentrations correlate with fat mass and leptin receptors are expressed in adipose tissue, suggesting that leptin regulates adipose tissue metabolism through autocrine signaling (14). However, the existence of variations in leptin concentrations in subjects with similar adiposity levels, among other evidences, have suggested that other factors, aside from adiposity, may regulate its secretion. Thus, the effect of leptin on adipose tissue metabolism does not appear to be entirely autocrine and it may also be mediated through the peripheral nervous system (14). Zeng et al. identified the neuro-adipose junctions that mediate the lipolytic effect of leptin, establishing that the leptin induced lipolysis is mediated by sympathetic neurons (15). It seems that, in prepubertal children, the peripheral nervous control of metabolism may be more important and, with age and its associated increase of fat mass, the autocrine effect of leptin is getting relevance. Moreover, a sex dichotomy in the sensitivity to central leptin has been demonstrated in rats, which has been associated with gonadal hormones (16).

It is known that the primary role of leptin in adipose tissue metabolism is the regulation of fat stores by controlling lipolysis and lipogenesis (11), as occur in adults. Thus, the negative association between leptin levels and NEFA concentration found in prepubertal children in our study is surprising. In this sense, similar to our findings, the Ulm Birth Cohort Study, analyzing 8-year-old children, also showed that leptin was negatively associated to NEFA concentrations (13). The authors suggest that, at this age, leptin seems to be related to energy providing processes in the fasting state and highlight the involvement of leptin in the regulation of fatty acid oxidation. They suggest that the inverse relationship between leptin and NEFA concentrations could be explained by the fact that NEFA are taken up by tissue for oxidation induced by higher CPT-1 activity stimulated by leptin (13).

Another additional explanation for our results may be related to the fact that epigenetic factors and differential gene expression patterns are associated with variations in the concentrations of leptin (17). In fact, in a recent publication, a different expression level of the FTO gene, extensively described as linked to obesity, has been related to the concentration of hormones produced by the adipose tissue, being positively correlated with leptin (18). Moreover, higher FTO expression has been associated with an increase of the concentration of FFA, suggesting that the FTO gene may play a role in the development of the impairment of glucose-lipid metabolism related to obesity in children (19). Unfortunately, due to the design of our study, we are not able to perform the analysis of gene expression levels in our samples.

We should mention the lack of information regarding Tanner stage, preventing us from using this datum as covariates in our analysis, as the main limitation of our study. As additional limitations, we should mention the lack of information on other adipokines in our prepubertal cohort, as well as the fact that, due to the design of our study, we are not able to perform the analysis of gene expression levels.

In summary, our study describes the influence of leptin on the association between obesity and NEFA levels in children, showing that, at the prepubertal age, leptin levels seem to explain the significantly lower NEFA concentrations observed in girls with obesity. Our study confirms the hypothesis of a role of leptin in the association between obesity and its related alterations, reporting a factor that may contribute to explain the described differences in the presence of obesity-related alterations by age.

## Data Availability Statement

The raw data supporting the conclusions of this article will be made available by the authors, without undue reservation.

## Ethics Statement

The studies involving human participants were reviewed and approved by Clinical Research Ethics Committee of the IIS-Fundación Jiménez Díaz. Written informed consent to participate in this study was provided by the participants' legal guardian/next of kin.

## Author Contributions

CV-V performed the research and contributed to data analysis. HO-S made important contributions to the interpretation of data. OD and IP-N performed the research. LS-G and TG-P contributed essential tools. CG designed the study, analyzed the data, and wrote the draft of the manuscript. All authors have read and approved the final manuscript.

## Funding

This work was supported by the Instituto de Salud Carlos III (grants number PI 18/01016 and FI 19/00180) and Biobank (grant number FEDER RD09/0076/00101). The funders had no role in study design, data collection and analysis, decision to publish, or preparation of the manuscript.

## Conflict of Interest

The authors declare that the research was conducted in the absence of any commercial or financial relationships that could be construed as a potential conflict of interest.

## Publisher's Note

All claims expressed in this article are solely those of the authors and do not necessarily represent those of their affiliated organizations, or those of the publisher, the editors and the reviewers. Any product that may be evaluated in this article, or claim that may be made by its manufacturer, is not guaranteed or endorsed by the publisher.
